# Client-Identified needs and agency-provided services at a harm reduction community based organization in the District of Columbia

**DOI:** 10.1186/s12954-015-0051-4

**Published:** 2015-06-03

**Authors:** Allison O’Rourke, Monica S. Ruiz, Sean T. Allen

**Affiliations:** Milken Institute School of Public Health at the George Washington University, 950 New Hampshire Ave, Suite 300, Washington, DC 20052 USA

**Keywords:** Harm reduction, District of Columbia, Case management, Social services

## Abstract

**Background:**

Harm reduction case management relies on client-identified goals to drive the provision of care in order to “meet clients where they are at”. This research measured the level of agreement between client-identified needs and agency-provided services at a community based organization (CBO) in Washington DC by examining: (1) the services clients most often identified, (2) the services most often given to clients by the CBO, and (3) the level of alignment between client-identified needs and services provided.

**Methods:**

Case file reviews were completed for 151 clients who received case management services at the CBO between January 2010 and February 2011. Client-identified needs and agency-provided services were extracted from case files and divided into 9 matching need and service categories: pharmaceutical assistance (e.g., prescription renewal), medical or dental care, housing, mental health services, substance use services, support services (e.g., support group meetings), legal assistance, and employment/job training. Client-identified needs and services provided were analyzed using McNemar’s Chi-square to assess for significant differences in discordant pairs.

**Results:**

Clients were mostly Black (90.7 %), heterosexual (63.6 %), HIV positive (93.4 %), and over 40 years old at the time of intake (76.2 %). On average, clients identified 2.44 needs and received 3.29 services. The most common client-identified needs were housing (63.7 %), support services (34.3 %), and medical/dental care (29.5 %). The most common agency-provided services were housing (58.2 %), support services (51.4 %), and medical/dental care (45.2 %). In 6 of the 9 service categories, there were statistically significant (p < .01) differences between those who received services not asked for and those who did not receive asked for services in the categories of pharmaceutical assistance, medical/dental care, substance abuse services, support services, legal assistance, and food access. In each of these matched service categories, the percentage of clients who received services not asked for was significantly higher than those who did not.

**Conclusion:**

This research shows that, while there is general alignment between the services that clients most often want and the services most often provided, there are still instances where services are requested but are not being provided.

## Background

Vulnerable populations utilizing community-based organizations (CBO) for service provision often have multiple basic needs that are unmet. These needs -- which include stable housing, food assistance, and medical care -- have been well documented in homeless or marginally housed [1, 2], substance using [3, 4], mentally ill [5], and HIV positive populations [6, 7]. Addressing these needs may lead to improved mental and physical health outcomes (e.g., stabilization of mental health and chronic health disorders) for individuals and decreased financial burden to the community (e.g., through primary care and emergency room visits) [8, 9]. Not addressing these needs can result in further destabilization and increased vulnerability. Often the hardest part of addressing these unmet needs is determining the best strategy to provide sustainable assistance, particularly for those individuals struggling with addiction or mental illness.

The harm reduction framework has been shown to be effective in addressing the needs of marginalized substance using and mentally ill populations. The primary tenet of the harm reduction philosophy is to “meet clients where they are at”. Under this tenet, providers assist clients in addressing and improving their health and well-being without asking them to change behaviors (typically, drug use) that they may not be ready to change. Originally, harm reduction was used to define the services provided to substance users in order to decrease their health risks while using illicit drugs. Multiple research studies have shown the effectiveness of syringe distribution and exchange in reducing the incidence of HIV/AIDS, reducing injection related practices which can lead to HCV and HIV, and serving as a “bridge” to further services when clients are ready for substance abuse treatment [10–16]. In the past decade, harm reduction as a method of reducing health risk has been expanded to include other public health fields including housing [17], smoking [18–20], and mental health care [21, 22].

Included in the recent expansion of harm reduction practices to other fields has been its application to case management provision. Previous research has demonstrated case managers’ and practitioners’ willingness to apply the harm reduction framework to service provision as an alternative to more rigid abstinence-only models of care [23–25]. Understanding needs from clients’ perspectives is a critical step in successful case management that may lead to positive, sustainable client outcomes [26]. In her discussion of harm reduction as a case management strategy, Odo states that engaging clients in an assessment about their needs allows the client to feel invested in their own case management process, which enables clients to be their own agents of change rather than passive recipients or reactors to imposed change [27]. This feeling of ownership of the change process is important: people, regardless of their station in life, want to be heard.

While the importance of client self-identification of need is well understood, less is known about how clients’ self-stated needs translate into actual provision of services. In order to better understand the relationship between client-identified needs and service provision, case management data were examined from a harm reduction CBO in the District of Columbia (DC) in order to determine the level of agreement between client-identified service needs and case manager provided services. This research sought to answer three questions: 1. What services did clients most often self-identify as needing? 2. What services did the CBO most often provide?, and 3. What was the level of alignment between client-identified needs and the services that were provided?

## Methods

A case file review was completed of 151 clients who received case management services at a harm reduction CBO in DC between January 2010 and February 2011. The CBO examined in this research was a secular, non-profit group that was supported primarily by funds received from private donations and philanthropic organizations. The CBO was widely recognized by the community as a safe place for individuals struggling with drug addiction to receive harm reduction services, including clean syringe distribution, HIV testing, sobriety support groups, and case management.

The research team completed a case file review on 100 % of the case management records from the CBO. Documents in these files included case manager notes, needs assessment forms completed by clients, and forms completed by other health care and social services professionals. Data points abstracted from the case files included: client demographic characteristics, mental health issues and needs, substance use-related issues and needs, medical history (including history of prescription drug needs, health conditions and co-morbidities, history of engagement in care, etc.), client-identified goals for engaging in services, and referrals and services provided by the agency. No personally identifying information was captured in the extracted data. Client case files were identified by unique identification codes that were assigned by the CBO at time of client registration and case file initiation. Extracted data were entered directly into a Microsoft Excel database. After data were initially entered, a random selection of 10 % of the case files was identified to be reviewed again by a different individual than the person originally reviewing the file to ensure uniform data extraction.

During the initial case management intake interview, clients worked with their case manager to identify up to three primary goals of their engagement with the CBO. These goals were extracted word for word from the client files during the review process and then grouped into nine different dichotomous (yes/no) client-identified service need areas: pharmaceutical assistance (e.g., prescription renewal, lost prescriptions, paying for prescriptions), medical or dental care, housing, mental health services, substance use services, support services (e.g., support group meetings), legal assistance (including court orders pertaining to overdue bills), and employment or job training. Each goal could potentially identify more than one service need area. For example, a client who indicated that he needed help getting a prescription refilled but also needed to see a doctor in order to have the prescription renewed would be categorized as needing both pharmaceutical assistance and medical or dental care.

Service provision was defined as any action taken by the case manager to assist the client in reaching their identified need. These actions included locating a requested service in the community if the need could not be addressed by the CBO directly and assisting the client in engagement with the new provider (e.g., completing necessary applications, etc.), directly providing the service to the client (e.g., giving the client access to the CBO’s food pantry), and scheduling/attending appointments for/with the client with other providers. Using the same nine service categories and coding method described previously, each service provided by the agency was coded dichotomously (yes/no) as it related to characterizing service provision. To follow on from the example above, if the service provider helped the client make an appointment with a doctor but did not follow up after the appointment in assisting with filling the prescription, the services provided would be indicated as medical or dental care but not pharmaceutical assistance. Service needs that were identified by the client were categorized as “client-identified needs”, and services that were provided to the client were categorized as “agency-provided services”.

In keeping with the harm reduction framework, client-identified needs and agency-provided services were compared in each of the nine service categories. A match occurred when a client-identified need in a particular service category was also indicated as having been addressed by the service provider in the same category. A discordant pair was identified when either (a) a client-identified a need in a service area but did not receive services in this area from the CBO or (b) a client did not identify a need in a service area but did receive the service from the CBO. McNemar’s Chi-square test was used to assess the relationship between concordant and discordant pairs in each of the nine service categories using each case files client-identified need and agency-provided service as a paired observation. The use of McNemar’s Chi-square test in this research was consistent with existing social work research methodologies for these types of data [28].

SAS 9.3 was used for all analyses

## Results

Client demographic and case file characteristics are shown in Table 1. In general, clients were Black (90.7 %), heterosexual (63.6 %), HIV positive (93.4 %), identified as male (51.0 %), and over 40 years old at time of intake (76.2 %). The primary reported routes of HIV infection were heterosexual (35.2 %) and male-to-male (MSM) sexual contact (21.1 %). Of the 139 clients with substance use information, 130 (93.5 %) reported having a substance abuse history and 95 (62.9 %) reported current substance use. Of the 95 clients reporting current use at time of intake, cocaine (*n* = 61), alcohol (*n* = 57), and heroin (*n* = 37) were the substances most often reported. Of the 151 clients, 96 (63.6 %) reported having at least one diagnosed mental health disorder; of these individuals, 26 % reported having more than one mental health diagnosis. Depression (72 %), bipolar disorder (22 %), and generalized anxiety disorder (9 %) accounted for the majority of reported mental health diagnoses. During the time period in which the client engaged with the service provider, 59.6 % had been or were currently involved in a program designed to assist HIV-positive individuals with adherence to medications and clinical care.

**Table 1:** Harm Reduction Organization Client Demographics (*N* = 151)

	Percent or mean (range)
Gender	
Male	51.0 %
Female	38.4 %
Transgender	10.6 %
Sexual Orientation	
Heterosexual	63.6 %
Homosexual	13.9 %
Bisexual	9.3 %
Other	7.3 %
Missing	5.9 %
Race	
Black	90.7 %
White	2.7 %
Other	6.0 %
Missing	0.6 %
Age at Intake	
Less than 30 years old	5.3 %
30-40 years old	18.5 %
41-50 years old	41.1 %
Over 50 years old	35.1 %
Employed	8.6 %
Current mental health diagnosis	63.6 %
Currently taking a mental health medication	32.5 %
History of Substance Use	93.5 %
Current Substance Use	62.9 %
HIV Positive	93.4 %
HIV Transmission Mode	
Heterosexual sex	35.2 %
MSM	21.1 %
IDU	12.0 %
Heterosexual Sex and IDU	7.0 %
MSM and IDU	1.4 %
Unspecified sex	2.1 %
Other	5.6 %
Missing/Unknown	15.6 %
Participated in HIV medication adherence program	59.6 %
Number of client-identified needs (mean)	2.44 (1–4)
Number of services provided (mean)	3.29 (0–9)

Of the 151 case files reviewed, 4 case files did not have any specified goals and were removed from all analyses going forward, leaving 147 case files. Table 2 shows the nine service categories with client-identified and agency-provided service provision rates. On average, clients identified 2.44 needs (range: 1–4) and received 3.29 services (range: 0–9). Overall, for all clients presenting for services, the most frequent client-identified needs were housing (63.7 %), support services (34.3 %), and medical or dental care (29.5 %). The most frequently provided services across all clients presenting for services by the CBO were housing (58.2 %), support services (51.4 %), and medical or dental care (45.2 %).

**Table 2:** Client-identified needs and services provided (*N* = 147)

Service Area	Service was not provided Count (%)	Service was provided Count (%)	Total Count (%)	Percentage Difference (% Clients who did not identify need – % Clients who did not receive service)	Percentage of services provided to clients identifying a need
Pharmaceutical					
Client did not identify need	88 (60.3)	38 (25.9)	127 (86.4)	20.4 %*	60 %
Client-identified need	8 (5.4)	12 (8.2)	20 (13.7)		
Total	96 (66.0)	50 (34.0)			
Medical or dental care					
Client did not identify need	66 (45.2)	37 (25.3)	107 (70.6)	15.8 %*	72.5 %
Client-identified need	14 (9.6)	29 (19.9)	40 (29.5)		
Total	80 (54.8)	66 (45.2)			
Housing					
Client did not identify need	38 (26.0)	15 (10.3)	53 (36.3)	−5.5 %	75.3 %
Client-identified need	23 (15.8)	70 (48.0)	93 (63.7)		
Total	61 (41.8)	85 (58.2)			
Mental health services					
Client did not identify need	97 (66.4)	19 (13.0)	116 (79.5)	6.2 %	66.7 %
Client-identified need	10 (6.9)	20 (13.7)	30 (20.6)		
Total	107 (73.3)	39 (26.7)			
Substance abuse services					
Client did not identify need	84 (57.5)	29 (19.9)	113 (77.4)	15.1 %*	78.8 %
Client-identified need	7 (4.8)	26 (17.8)	33 (22.6)		
Total	91 (62.3)	55 (37.7)			
Support services					
Client did not identify need	55 (37.7)	41 (28.1)	96 (65.8)	17.2 %*	68 %
Client-identified need	16 (11.0)	34 (23.3)	50 (34.3)		
Total	71 (48.6)	75 (51.4)			
Legal assistance (including delinquent utilities)					
Client did not identify need	105 (71.9)	22 (15.1)	137 (87.0)	8.9 %*	52.6 %
Client-identified need	9 (6.2)	10 (6.9)	19 (13.0)		
Total	114 (78.1)	32 (21.9)			
Employment/job training					
Client did not identify need	98 (67.1)	13 (8.9)	111 (76.0)	−6.2 %	37.1 %
Client-identified need	22 (15.1)	13 (8.9)	35 (24.0)		
Total	120 (82.2)	26 (17.8)			
Food access					
Client did not identify need	76 (52.1)	37 (25.3)	113 (77.4)	15.8 %*	57.6 %
Client-identified need	14 (9.6)	19 (13.0)	33 (22.6)		
Total	90 (61.6)	56 (38.4)			

*McNemar’s Chi-Square, p < .01

When looking more specifically at the number of clients who expressed need in each service category and how many of those clients received services from the CBO, the percentage who received services ranged from 37.1 % to 78.8 %. The three service category with the highest percentage of clients identifying the need receiving services were: substance abuse (78.8 %), housing (75.3 %), and medical or dental care (72.5 %) services. While the services with the lowest percentage of clients identifying the need and receiving services were: employment/job training (37.1 %), legal assistance (52.6 %), and food access (57.6 %).

Using McNemar’s Chi-square test, six service categories where identified with statistically significant differences in discordant pairs, i.e., services requested but not provided and services provided but not requested. These data are shown in Table 2. A statistically significant difference (20.4 %; *p* < .01) was identified between the 5.4 % of clients who requested assistance with pharmaceutical access but did not receive this service and the 25.9 % of clients who received pharmaceutical assistance but did not request it. Other significant (*p* < .01) discrepancies were found in the areas of medical care (9.6 % vs. 25.3 %, difference: 15.8 %), substance use services (4.8 % vs. 19.9 %, difference: 15.1 %), support services (11.0 % vs. 28.1 %, difference: 17.1 %), legal assistance (6.2 % vs. 15.1 %, difference: 8.9 %), and food access (9.6 % vs. 25.3 %, difference: 15.8 %). In each of these service categories, the percentage of clients who received unwanted services was significantly higher than those who did not receive services they had requested.

## Discussion

Addressing client-identified needs is a core component of the harm reduction case management framework. This research sought to better understand the agreement between client-identified needs and services provided by a harm reduction CBO. When looking specifically at those clients who identified a need and actually received services to address that need ranged from 37.1 % to 78.8 %. The categories with the largest percentage of clients identifying needs and then receiving services were substance abuse services, housing and medical and dental care. The categories with the lowest percentage of clients identifying a need and receiving service to address the need were employment, legal assistance and food access. We found that the most commonly identified client needs -- housing, support services, medical or dental care, food access, and substance abuse services – aligned with the most commonly provided services. However, for other client needs, there were not high rates of matching between the services requested and the services provided.

When taking these analyses one step further to look at discordance, we found that a large percentage of clients received services even when they did not express a need for those services. This provides us with some additional insight into the client - service provider relationship. One interesting fact that came from this analysis is that the CBO most likely had the capacity to provide certain services to clients but that, for some reason, these services weren’t necessarily being directed to the clients who asked for them. Unfortunately, we do not have the data to understand why certain available services were not provided to clients requesting them. Similarly, we do not have the data available to understand how not receiving requested services affected clients’ level of satisfaction or engagement with the CBO. Future research should focus on better understanding this disconnect between clients’ needs and services provided in order to enhance the quality of and satisfaction with service delivery.

It is not surprising that provision of housing was both the most frequently requested client-identified need and the most frequently provided service. There is a substantial and growing literature documenting the benefits of housing stability for marginalized populations [29–31] and strong evidence to suggest that provision of stable housing can benefit recovery for those dealing with mental illness [32], substance abuse [33, 34], and co-occurring disorders [35]. While the majority (75.3 %) of clients who identified a housing need received assistance with housing from the CBO, a measure of the stability of the housing provided was not available. Given the breadth of research linking stability in housing to positive outcomes in other areas, future work should examine the stability of the housing that was provided to clients and the impact that stable housing has on CBOs’ ability to address clients’ other identified areas of need.

Service availability and provision may also be dependent on client-specific factors. In our study, the majority (93.4 %) of clients were HIV positive, which gave them enhanced access to certain types of services. For example, specific funding is available in DC for coverage of medical care and pharmaceutical access for individuals living with HIV; these resources are not available for those who are uninfected. This variability in available services may have skewed the way in which case managers were able to provide services, prompting them to provide services that the client did not request simply because those services were available to the client on the basis of their diagnosis. Needless to say, this causes a disparity in available resources for uninfected individuals that would not be easily remedied unless those individuals were to seroconvert. Future research is needed to examine how clients perceive and understand the barriers that may be in place that limit their access to necessary resources and services, and how case management service providers address these perceptions in the course of addressing clients’ needs.

Service provision may not only be constrained by availability within the community, but also by factors that are specific to the service providing organization(s). For example, financial constraints on CBOs’ service provision can result from fiscal limitations, such as budgets that are not large enough to meet program needs or funding that is earmarked for provision of specific services (i.e., grants which specify how monies can be spent). Operational constraints within the CBO may also hinder service provision. For example, there may be an insufficient number of support staff within the organization to handle the client caseload, or there may be rules in place that require clients to show stabilization in one area before being allowed assistance with another (i.e., sobriety before being able to obtain job training). Further research – particularly prospective studies – are needed to examine these barriers and their impact over time on client service provision, and to understand how changes in these organizational-level factors may influence client satisfaction with and retention in case management.

Other significant constraints in addressing clients’ needs may be attributable to the nature of the resource and service infrastructure itself. Since case managers are required to work with the programs that are available in their community, they do not have the ability to modify the policies or practices that may restrict their clients’ ability to access or participate in services. In the case of our study, we found that some of the most common barriers to the immediate provision of services included inability of the client to prove DC residency (e.g., no legal identification), proof of sobriety (e.g., clients needing proof of a recent negative drug screen), and evidence of positive HIV status (e.g., clients having no medical records to verify diagnosis). Without first assisting clients in obtaining the documents needed to verify access to services, case managers’ hands are often tied in providing services until these requirements can be met. These limitations curb CBOs’ abilities to address the many needs of their clients let alone follow a client self-identified order of prioritization, as the harm reduction framework would recommend. Additionally, case managers are often burdened with a heavy caseload and, therefore, are not able to immediately address each client’s identified goals.

In 6 of the 9 service categories, more clients received unwanted services compared to clients who received requested services. While this is interesting, there are no data pertaining to what level of agreement or disagreement in case management is consistent with compliance to harm reduction principles. Nonetheless, given the multiple needs of the population and the potential benefit that could come from having those needs addressed, it may be reasonable to assume that compliance with a harm reduction framework would allow for ongoing improvement in CBOs’ abilities to meet client-identified needs. Building on this research, it would be interesting to follow the provision of services at a CBO in which the provider was able to self-assess on how well they felt they were able to apply the harm reduction principles and what obstacles they felt kept them from following the framework more completely.

This study had several limitations. First, the data abstracted from client case files were limited to what was documented by case managers. Many of the case files had little information other than a listing of services that were provided or offered. The variability in available information limited our ability to assess other aspects of the client-CBO encounter (such as strong rapport with a case worker) that may have affected clients’ outcomes. Second, most of the clients were in need of a range of services and, in all likelihood, were simultaneously seeking services at other CBOs. Therefore, our data may not provide a complete picture of which services were needed, which service needs were met, and whether or not other CBOs were able to provide clients with resources. A third limitation pertains to the variability of CBOs’ ability to provide specific services, either because of funding constraints or lack of available community resources. This variability may have affected the degree to which the CBO was able to address clients’ needs in a timely and consistent manner. Lastly, client-identified needs were based on the goals that clients created with their case manager at the time of intake. The CBO limited clients to listing only three goals. While these goals could identify more than one service area and, on average, clients identified less than three service areas, this is still a limitation. Ideally, client need identification would utilize a more comprehensive list of available services and resources that the client would then review, checking the services that he or she desires. Based on this checklist, the case manager could work with the client to identify the priority needs and then work to address those needs. Future research may be needed to develop better, more standardized methods for assessing and recording clients’ service and resource needs, as well as tracking how well and how promptly those needs are addressed by service providing organizations.

## Conclusion

In conclusion, this research shows that, while the service categories that clients most often identify needing and the services most often provided align, these services are not necessarily being delivered to the individuals who request them. In a harm reduction framework of case management, it is important to allow client-identified goals to drive the provision of care received from the case manager in order to build a strong and lasting relationship. Future research should seek to understand the barriers – from both the client and service provider perspectives -- that may be involved in preventing clients from receiving services in identified areas of need. Additional work is needed on the development of reliable measures of adherence to harm reduction strategies for service provision; this would help organizations serving vulnerable populations to better evaluate their effectiveness in addressing clients’ needs in a manner that is respectful of individuals’ autonomy and agency as they engage in care and services.
